# Neuralgia and Its Treatment

**Published:** 1880-05

**Authors:** Truman W. Brophy

**Affiliations:** Chicago.


### ARTICLE III.

### *Neuralgia and its Treatment.*

BY TRUMAN W. BROPHY, D. D. S., CHICAGO.

[Read before the Wisconsin State Dental Society, at Milwaukee July 16, 1879.]

The term neuralgia, which stands as the title of the paper I now have the honor of presenting to you, is derived from the Greek roots, "Neura," a nerve, and Algios, pain; henoe, we have, when translated, nerve-pain, or pain in a nerve. "Under the head of Neuralgia," says Prof Flint, "are embraced a group of local affections characterized by pain occuring without inflammation, or any appreciable change in the parts affected. All that can be said of the pathological character of these affections is, they consist in a perversion of sensibility." Accepting this as the correct definition of *true* neuralgia, we have to consider another species of nerve-pain, induced by local irritation, the result of which is an engorged condition of the capilliaries, and their pressure against the nerve-sheath produces pain. It is this species of neuralgia, which the dental practitioner is most frequently called upon to treat.

As the result of investigation by Tadcliff, an English scientist, a nerve was found to be analogous to the pole of an electric battery. The nerve-sheath acting as the negative pole, while the nerve proper acts as the positive. It is by this means he accounts for the transmission of sensation, from the periphery of a nerve, to its ganglion, thence to the seat of sensation. Although neuralgia is the cause of suffering of the greatest severity, it is not, strictly speaking, a disease, neither do the most intense and long continued attacks terminate fatally. Patients are, in many instances, subject to neuralgic pains during the greater portion of their lives, and finally die of diseases which were not induced by, nor in any way connected with neuralgia. According to an analysis by Valleix, a French physician, as to the manner in which neuralgia terminates, he found, that in seven-ninths of the cases examined a permanent cure took place. In one.ninth there was improvement without recovery, and in the same proportion there was neither cure nor improvement.

There has been considerable speculation among investigators, as to the cause of neuralgia, when it cannot be ascribed to any apparent lesion ; foremost among whom is Dr. Francis Edmond Austie, who claims that true neuralgia *is* a disease. In the third chapter of his monograph on this subject (p.110) he says, " I expect to convince most readers, that the essential seat of every true neuralgia is *the posterior root of the spinal nerve, in which the pain is felt*, and that the essential condition of the tissue of that nerve-root is *atrophy*, which is usually non-inflammatory in origin." The comments of the best authority, however, admit that such a condition is met with, but regard it as an explanation of peripheral pain. I fail to find any one willing to support Dr. Austie's theory of atrophy of the posterior root of the spinal nerve.

It is not my intention to comment on the causes of *true* neuralgia to any extent, but believing that lesions within the oral cavity are the predominating causes of neuralgia, and that it should receive more attention and be thoroughly understood by us, as in my opinion the oral or dental surgeon is, owing to the very nature and location of the disorder, the proper one to treat it. I shall attempt, therefore, to point out some of the causes of

TRIGEMINAL NEURALGIA, and its treatment. In doing so I shall advance no new theories, for I have none, but shall relate a few cases which I believe to be of deep interest to us as a profession, and which have not heretofore come to your notice.

There are few persons affected with neuralgia, as the term is generally understood, that seek the services of the practitioner of dentistry to obtain relief. When a patient thus affected presents himself to us, he usually denominates his trouble "toothache," and believes that the removal of a certain tooth, or teeth, will effect a permanent cure; when in fact, the teeth in some instances are in no way implicated, the pain emanating from other causes. It is in the treatment of such cases that the best of judgment is demanded, and the ability of the practitioner put to the test, to diagnosticate with the accuracy to be expected from one professing to possess the requisite knowledge of pathology and therapeutics, to treat with reasonable success these affections, the causes of which are sometimes very obscure, and which are frequently, erroneously attributed to the teeth. All members of our profession have no doubt seen persons whose teeth have been removed by advice of their physician, with a view of being relieved from general neuralgia. The teeth of these persons were pretty good, none indeed being sufficiently carious to produce odontalgia. We have observed, that in some such cases the result desired was not obtained, the pain not being abated even in the slightest degree. On the contrary, the removal of the teeth has been to the patient a serious and irretrievable loss, all of which must be regarded as the result of failure, or inability, to correctly diagnosticate the condition.

On the other hand we have seen persons, who have been under treatment for neuralgia for months, and in some instances years, taking medicine almost daily, and yet obtain only temporary relief, if any; when upon close examination of the teeth, removing all the fangs which are not sufficiently firm to be crowned, and properly treating and filling such teeth as can be preserved, the neuralgia quietly, and in many caaes effectually passes away. The exciting causes of *true* neuralgia are usually a damp, cold atmosphere, miasmatic influences, or an anæmic condition of the system; to this we might add syphilis. As before stated, the species which we, as a profession, are most frequently called upon to treat, emanates from some lesion, or the presence of some substance which impinges upon the nerve-sheath, thus causing pain.

Of these causes, by far the most common is nervous odontalgia. However, it may proceed from periostitis, exostosis, the contracted arch and crowded teeth, partial calcification of the pulp, pulp-nodules, or fractures of the maxillary bones, causing spiculæ of bone to come in contact with, and irritate the nerve. This latter condition may, and doubtless does result in some cases, from the unskillful use of the forceps. Neuralgia may be induced by a cicatrix, which contracts the soft tissues adjacent to the nerve, thus producing pressure and consequently pain.

Indeed, it may be caused by anything which interferes with the organic functions of life. What, then, shall the first step be to alleviate our suffering patients, from this dreaded and terrible affection? Answer: search for, appreciate and remove the cause; and since trigeminal neuralgia originates most frequently from carious teeth, our attention should always be directed to a thorough diagnosis of the mouth. If the teeth present an appearance of soundness, be not satisfied with this, as there are many cases where caries are proximately situated, and so obscure that time is required to secure sufficient space to accurately diagnose. Some will doubtless say, why do you speak of odontalgia or exposed nerve as the origin of neuralgia? Why do you confound the terms? or how is odontalgia distinguished from neuralgia? In answer, I will say, the sensations produced by neuralgia are the same and identical with nervous odontalgia.

We have all been consulted with reference to pain, described by our patients as being of the neuralgic type, periodical and lancinating. The patient is unable to locate it, but imagines it may possibly proceed from the teeth. A close examination reveals, along the surface of a tooth adjacent to its neighbor, and in the majority of cases just about the free margin of the gum (which is absorbed) and underneath the enamel, carious dentos; this may or may not be broken down. The demineralized tooth-substance at this point may be in its perfect original shape, but by probing it is found to be a soft, gelatinous mass, which easily allows infiltration of the secretions of the mouth, hence the source of pain. Such cases are most frequently observable in patients of middle or advanced age. In case fractures of the bone have occurred, a free incision should be made and the disconnected bone removed. Some of the most aggravating cases of neuralgia are due to fractures of the bones, necrosis following, with a disposition of new or secondary bone, which so involves the sequestrum that the efforts of nature cannot expel it.

The skill of the surgeon, therefore, must come to the rescue. The effect of the so-called pulp-nodules and partial calcification of the pulp upon the nerves supplying the teeth is familiar to all. We realize the necessity of relieving the suffering, and yet, our task is one surrounded with difficulty of the most complicated nature, more especially so when the patient is unable to locate the seat or origin of the pain. To diagnose correctly the cause of neuralgia, when due to pulp-nodules, when no particular location of the pain can be designated by the patient, is a task worthy of the most profound observer, and the most thorough and accurate diagnostician. Fortunately, however, cases where the pain cannot be located are rare. The teeth containing these excresences or nodules within the pulp-canal, are usually sensitive to the tap of an instrument. The pain is increased by quick changes of temperature, or upon hard pressure. The irritation of the tooth-pulp and the dental periosteum, occasioned by these nodules, is followed by an elongation of the tooth, sometimes slight, but distinct. This is probably the most marked symptom of the disturbance in question. These nodules doubtless are produced by the odontoblastic membrane which has been subjected to gradual, persistent, prolonged, though slightly irritating influences.

The treatment for this source of nerve pain is, of course, to remove the cause, and that can be accomplished by drilling to the pulp canal and carefully removing the little, pebble-like, ossific bodies, with a very fine pointed excavator. If, however, the pain experienced in their removal is very severe—as it generally is—the arsenious paste may be applied, after first reducing the congestion of the blood-vessels, then the nodules can be painlessly removed and the roots treated according to the approved modes of the day.

*Exostosis* of the fangs of the teeth, a fruitful source of neuralgia is, indeed, as difficult to diagnosticate as pulp-nodules, unless for fangs affected and enlarged to that extent which clearly locates and defines the affection. In exostosis, as in pulp-nodules, it is gratifying to say that the pain is more localized than diffused.

The sensation produced by dental exostosis is that of fullness, and it is when the alveolus does not yield by absorption, in proportion to the growth of the tumor, that neuralgia is produced, sometimes of the most painful type, and if not understood, it may be treated an indefinite time without any beneficial effects.

My experience and observations teach me that exostosis results, in a very large majority of cases, from irritation of long standing, and final death of the pulp. Sometimes, it occurs upon the fangs of perfectly sound teeth, but, far more numerous are the cases where a portion, or the whole of the crown of the tooth, has been destroyed by caries. A sound tooth, affected with exostosis, not always, but usually, has characteristics differing in a marked degree from its fellows, and it is through this medium the offending member is pointed out. I allude to the consolidation, in the whole tooth-structure, of calcium salts which renders the resisting force of the organs, to the instruments, greater than is ordinary, and far in excess of the adjacent teeth. This condition can usually be recognized by the experienced eye.

The treatment for neuralgia resulting from exostosis is, to remove the affected tooth. Some of our professional brothers, with pride in preserving such teeth, might extract it, remove the tumor and replace the tooth. The fears of tetanus, however, and a vivid recollection of the loss of a patient of this disease, by a professional friend, who had replaced teeth, after removing the exostosis, would debar your essayist from such a practice.

Mr. Salter, in his admirable work on "Dental Pathology and Surgery," describes a case of intense neuralgia of the eye-ball and face, which caused a change in the color of the iris, resulting from carious teeth.

This case was indeed a remarkable one. It is so important to us, and illustrates how men of the highest medical skill sometimes fail in making a correct diagnosis of lesions, or affections, which have their origin in the teeth, that I shall quote it. He says, "Mrs. C., aged about thirty years, was sent to me by Dr. Oldam on the 21st of June, 1867. She was suffering from neuralgia of extraordinary severity, affecting the left eye-ball, and the left side of the head and face. She had suffered almost continuously for ten years, the attack commencing on her recovery from a bad confinement; and no medicines had given her distinct relief. The most singular and interesting part of the case is, that the iris of the affected eye has completely changed color under the influence of the continued pain. Both her eyes were originally of a deep and bright hazel color; the iris of the right eye remains as it was, but the left has changed to a dull gray, without any trace of the original hue. This change has occured progressively till the eyes are now as if belonging to two people of totally different complexion. No artificial painting could exhibit a more strange and striking contrast. When this lady came to me she was in a pitiable condition, suffering such agony as I have seldom witnessed, and worn out and exhausted by the continual sleeplessness which the pain had occasioned. Upon examining her mouth, I found that the left lower dens sapientia and the first upper bicuspid were badly carious. I removed these teeth, and the operation was attended by a terrible paroxysm of neuralgia, but upon recovery from this, the patient expressed her conviction that the extraction of the teeth had cured her, and so it proved. From that day till the eighth of October, three months, she was entirely free from pain; she had scarcely known such immunity, even for three days, since her neuralgic suffering commenced, ten years before. As she expressed it, 'it was like a new life to her." The only comment I wish to make upon Mr. Salter's treatment of this case is, I believe he would have done the lady a better service had he treated, and, if possible, preserved these teeth.

He continued: "On the eighth of October the old pain came back again, and my patient came to me on the tenth, just in the condition she had been at first. I now found that the second upper bicuspid was carious, and intensely tender. Upon its removal a considerable exostosis was seen on the root. The pain vanished with the tooth, and the patient wrote to me, a week after, saying she was quite well. The iris of the left eye, however, has never gone back to its original color, but remains a leaden gray. The eye does not appear to have undergone any other nutritional change: its vision has not been affected."

A case of neuralgia, of great importance to the dental surgeon, was recently described to me by Dr. Deering, of Chicago. A lady about forty-eight years of age, strong and healthy, residing in Kansas City, called on a dentist of that place to be relieved from severe neuralgic pain, described by her as analogous to toothache; she had lost all her superior teeth about twenty-five years earlier, and had since worn artificial teeth. The pain was located on the left side of the arch, in the region of the antrum. The dentist examined her mouth carefully, and concluded that the plate she was wearing was the sole cause of her suffering.

The lady at once had a new plate constructed by him, but her suffering was not in the least abated. The dentist thereupon called a surgeon, who also examined her mouth, and pronounced her condition the result of a tumor upon the palatine plate of the left superior maxillary bone, and advised an operation. She consented. The mucous membrane was divided, a chisel and mallet were brought into requisition, and an effort made to remove the tumor. Having no success, however, he desisted, feeling he was not equal to the case.

The following day the face was greatly swollen. The surgeon assured her that her's was an unusal case, and advised her to go at once to Philadelphia, and employ a distinguished surgeon of that city, who would effectually cure her. Following his advice, she went to Philadelphia, via Chicago.

Stopping here a few days with friends, she was requested to call on a dentist for his opinion.

He examined the case and informed her that she was on the right track. It was a very serious matter and required the highest skill to treat it. On arriving in Philadelphia, she consulted the eminent surgeon, and he pronounced the trouble ulceration of the mucous membrane of the antrum. With a drill he made an opening into the antrum, and through the opening applied the usual remedies for this disease. This was continued for some time with constitutional treatment, consisting af quinine, iron, and other remedies which were indicated.

This treatment was continued eighteen months, with very little, if any relief. She left Philadelphia to return home, with directions to continue the quinine two months longer, and report by mail.

She stopped in Chicago, on her return home, and called on Dr. Deering, who was the first to correctly diagnosticate her case. The pain was still very severe, and located in the region of the antrum. A protuberance was observed midway between the alveolar ridge and the palatine-suture of the maxillary bones. An exploring instrument was passed through to the enlargement, and an idea formed by the doctor of the nature of the trouble.

He then questioned her in regard to her dental history. She informed him that her teeth were all extracted when she was about twenty-five years of age.

The question was then asked, " Are you sure they were *all* removed ?" It then occurred to her for the first time that on each side of her mouth the second deciduous molar remained at that time, the dentist having called her attention to the fact, and remarked that possibly she would sometime get the second bicuspid teeth. An operation was performed, a bicuspid tooth was found imbedded lengthwise in the maxillary bone, with apex well into the antrum. This was with some difficulty removed. The crown was chipped off a little by the Kansas City surgeon, who failed to remove it, otherwise the tooth was in a perfect condition. After the effects of this operation had passed away, the patient, for the first time in four years, had perfect immunity from pain. Three months subsequently, however, pain of the same character was felt on the opposite side, and another operation was performed with the same result. Dr. Deering kindly brought this lady to my office, and upon examining the mouth, two slight grooves were seen on either side where the teeth were removed. No neuralgic pain has been felt since. The opening made into the antrum, however, has not closed. The inflammation brought on by the use of the strong medicinal applications is, indeed, difficult to reduce. No evidence of the operation, other than that spoken of, exists. Thus a patient suffered pain of the greatest severity during so long a period, all owing to failure on the part of the professional gentlemen she had employed to accurately diagnosticate her case.

The lesson to be learned from this case is, think of more than one thing when making up a diagnosis. This was a case belonging strictly to dental surgery, and had the dentist possessed sufficient knowledge to accurately diagnose the case, the difficulty could have at once been corrected. As before stated the cause of true neuralgia, is a question upon which the most eminent pathologists cannot agree.

It is sufficient for our purposes, however, without discussing the different theories advanced, to state that it is generally conceded, that true neuralgia results from some organic disturbance, as inactivity of the liver, and consequently constipation of the bowels; especially is neuralgia due to habitual constipation. In the plethoric patient, neuralgia may usually be attributed to the presence of uric acid in the blood, but this class of patients is not often subject to neuralgic affections. By far the most common cause of true neuralgia is anæmia; a pallid, pinched-up face; a dull, sunken eye, accompanied by poor appetite: such a person is exceedingly liable to neuralgic attacks.

Romberg, when speaking of anæmic conditions and neuralgia resulting therefrom, says, "It seems as if pain were the prayer of the nerve, for healthy blood." In the treatment of neuralgia resulting from this cause, the protracted use of tonics must be employed. If, however, the attacks of pain are very severe, they may be relieved by the use of narcotics. But permanent relief cannot be looked for until the system is built up. If the patient suffering from neuralgia resides in a malarious district, quinine is indicated. Lead poisoning is another cause of neuralgia, which I have no doubt has been caused in many instances by the unscrupulous dentist, who has repaired, or had repaired by a tinner, silver and other metalic plates, with soft solder, lead being the chief constituent.

An interesting case of trigeminal neuralgia came into my charge two years since. A gentleman, aged thirty eight, consulted me with reference to pain located by him in the left second superior molar. A careful examination revealed no caries, no accumulation of tartar, no periostitis, but slight congestion of the gums; also slight inflammation of the face at the terminal branches of the infra-orbital nerve.

My patient requested me to extract the tooth described. I informed him of its soundness, and refused to comply with his request, also informing him that his trouble was neuralgia. I applied Tr. Aconite to his gums, and prescribed as follows:

℞

Prusiate of Potass., - - - - ʒi.

Eau de Cologne, - - - - } aa ℥i

Tr. Aconite (Flemming's,) - - }

M. Sig.

Dilute, if too strong, with water, apply with sponge every two or three hours. I directed him to keep warm flannel constantly applied to the affected side, and report the following day, when I found his condition much improved, the paroxysms of pain occuring at greater intervals and less severe. I prescribed Sulphate of quinine grs. xxiv. in four doses, a dose every three hours, and to continue the local application.

These directions were carefully followed, and two days after he pronounced himself well. Five days after this, however, another paroxysm of pain returned, when he declared that I must extract the tooth, but I dissuaded him, and put him again on the same treatment, with the same result. I saw my patient on Friday, three days after the last administration of quinine, when the information of his recovery was with great pleasure received.

The cause of the neuralgia in this case was by no means clear to me, as the patient was unusually robust and strong, not being subject to any ills. During the subsequent week, however, light was thrown upon the case. My patient informed me that on the Sunday following his last conversation with me, a physician removed from him a tape-worm forty-eight feet in length. Then the cause of the disturbance of the nervous system, and consequently the neuralgic pain, was at once understood. The disturbance or agitation of the system in this case must have been very great.

The day subsequent to the operation, the local applications to the face were frequently resorted to, to control the occasional paroxysms of pain.

I saw this patient, during the past month, when I learned that no trace of neuralgia had since recurred. Another case of neuralgia, of great severity, came to me during the past week. A gentleman of sedentary habits, aged about fifty years. The pain in his case was in the terminal branches of the infra-orbital nerve, also in the supra-orbital. Finding no carious teeth, which would be liable to cause the disturbance, I questioned him in regard to his general health, occupation, &c. I found that he was subject to constipation of the bowels to such an extent that it was necessary to take medicine every morning.

The pain was steady in this case; it came on at about ten A. M. and would subside at about six P. M. He had suffered only a week, but he said it seemed like a month. The eye on the affected side (the right side) was greatly inflamed, and the tears were constantly flowing. The mucus, from the right nostril also flowed profusely. This I believed to be a case of *true* neuralgia, and I gave him the following:

℞

| | |
|---|---|
| Quinia Sulph. - - - - | grs. xxiv. |
| Acidum Sulphuricum (dil) - - - | ʒ j |
| Elixir Taraxacum, a. d. q. s. - - | ℥ ij |

Sig.

To be taken in four doses; one dose every three hours. He called two days subsequently and reported a perfect immunity from pain. I have not seen him since, and therefore believe it has not returned. A rule which I have adopted, is as follows: When a patient presents himself, whether suffering pain or not, to first carefully remove the salivary calculi and cleanse the teeth; then medicate those which require it, if any, then extract every fang in the mouth deprived of its crown, excepting, of course, those which may be restored to usefulness by mounting them with crowns. This done, I am prepared at the next sitting to make a more intelligent diagnosis for neuralgia or other affections.

We should firmly object to allowing our patients to dictate what we shall do If they wish our services, and have confidence in us, we should insist on doing what we know to be for their interest.

In writing on this subject, gentlemen, I may have wandered to some extent, and transcended the prescribed bounds of dentistry, according to the views of some; but if I have done so, it is my weakness, and I am not responsible for it. The all-absorbing question again persists in thrusting itself before us: Are we to treat surgically or therapeutically diseases of the teeth, and those diseases which emanate from the teeth? Are we to content ourselves with filling carious teeth, oblivious of the chemical action which brings about their disintegration, a recurrence of which *will* follow the most perfect manipulations, without even an effort on our part to correct it?

Shall our knowledge of surgery be confined to extracting teeth? Shall our occupation be that of plate-makers? Is our knowledge of pathology to be confined to congestion and inflammation of the dental pulp and gums? Shall we say our profession is conversant with the branches of medical science, materia medica and therapeutics; when creasote and arsenious paste are the sole stock of remedies in the hands of hundreds of men practicing dentistry in this country, and making local applications to inflammation or suppuration of the mucous membrane, when in many such cases no man can cure them without constitutional treatment? And even in this advanced and enlightened age, to see our professional brothers, presumably in the full possession of their reason, surrounded by books of reference of the highest order, and periodicals filled with the richest fruit of experience, observation and knowledge; practicing their profession after the ideas of the generations of the past? Or shall we not put forth a greater effort in acquiring knowledge of surgery, pathology and therapeutics, as far as they can, in any *way*, *shape* or *form*, be associated with, or applicable to, our specialty? My answer is, Yes. What the better men in the dental profession stand most in need of to-day is a greater, more extensive knowledge of the action of remedies, and how to administer or apply them.

I care not to be a general practitioner of medicine; my profession is a better one for a young man. The views of many are opposite to mine; but as for me, I do not care to suffer the humiliation of turning over to the general practitioner of medicine cases which are strictly within the province of dentistry, on the ground of not understanding them.

The great work to be accomplished by our profession is to prevent, if possible, caries of the teeth. If, however, we fail in this, our next duty is to arrest it. This, in my opinion, can only be accomplished by the aid of a knowledge of chemistry and therapeutics, a complete control of the patient, thus enabling the practitioner to prevent or correct a contracted arch and crowded teeth, a strict observance of the laws of hygiene, and last, but not least, the careful, trained and skillful hand of an honest, conscientious dentist. So far as we succeed in preventing and arresting caries of the teeth, just so far we shall be instrumental in doing the greatest service within our power to humanity, and nipping in the bud the condition which in some instances grows to such gigantic proportions, in the form of trigeminal neuralgia.—*Missouri Dental Journal.*

